# Amyloidβ Peptides in interaction with raft-mime model membranes: a neutron reflectivity insight

**DOI:** 10.1038/srep20997

**Published:** 2016-02-16

**Authors:** Valeria Rondelli, Paola Brocca, Simona Motta, Massimo Messa, Laura Colombo, Mario Salmona, Giovanna Fragneto, Laura Cantù, Elena Del Favero

**Affiliations:** 1 Dept. of Medical Biotechnologies and Traslational Medicine, University of Milano, LITA, Via F.lli Cervi, 93. 20090 Segrate (Milano), Italy; 2 Dept. of Molecular Biochemistry and Pharmacology, IRCCS Istituto di Ricerche Farmacologiche “Mario Negri”, Via La Masa 19, 20156 Milan, Italy; 3 Institut Laue-Langevin, 71 avenue des Martyrs, BP 156, 38000 Grenoble Cedex, France

## Abstract

The role of first-stage *β*–amyloid aggregation in the development of the Alzheimer disease, is widely accepted but still unclear. Intimate interaction with the cell membrane is invoked. We designed Neutron Reflectometry experiments to reveal the existence and extent of the interaction between *β*–amyloid (A*β*) peptides and a lone customized biomimetic membrane, and their dependence on the aggregation state of the peptide. The membrane, asymmetrically containing phospholipids, GM1 and cholesterol in biosimilar proportion, is a model for a raft, a putative site for amyloid-cell membrane interaction. We found that the structured-oligomer of A*β*(1-42), its most acknowledged membrane-active state, is embedded as such into the external leaflet of the membrane. Conversely, the A*β*(1-42) unstructured early-oligomers deeply penetrate the membrane, likely mimicking the interaction at neuronal cell surfaces, when the A*β*(1-42) is cleaved from APP protein and the membrane constitutes a template for its further structural evolution. Moreover, the smaller A*β*(1-6) fragment, the N-terminal portion of A*β*, was also used. A*β* N-terminal is usually considered as involved in oligomer stabilization but not in the peptide-membrane interaction. Instead, it was seen to remove lipids from the bilayer, thus suggesting its role, once in the whole peptide, in membrane leakage, favouring peptide recruitment.

The mechanisms underlying Alzheimer’s Disease (AD) are not completely understood, but genetic, pathological and biochemical observations indicate that the progressive production and accumulation of *β*-amyloid peptides (A*β*), proteolytic fragments of the membrane-associated amyloid precursor protein (APP), play a pivotal role^1^. Neurons release these peptides in a soluble form that progressively generates different molecular assemblies from oligomeric to multimeric structures, ending up to fibrillar aggregates. In particular, soluble oligomers are considered as the main responsible for the onset and progression of the cognitive dysfunction^2^,^3^. One of the peculiar properties of soluble A*β* oligomers is that, unlike mature fibrils, they are “membrane-active” species that can promote membrane puncturing and increase its permeability^4^,^5^,^6^. It has been observed that, upon interaction with A*β*-peptide oligomers, the membrane undergoes reorganization, with an increase of the lipid chains volume^5^ an expansion of the surface area and an alteration of microviscosity^7^,^8^,^9^. Recently, it has been reported that interaction with A*β* peptide modifies the morphology and mechanical properties of mixed model membranes containing cholesterol and sphingomyelin, depending on cholesterol content^10^. Involvement of cholesterol in brain diseases is recurrently claimed^11^. Within membranes, the interaction sites for either APP or A*β* are localized at those domains, such as rafts and caveolae^12^, enriched in cholesterol and GM1-ganglioside^13^,^14^,^15^. Lipid rafts constitute themselves endogenous seeds for A*β* aggregation. Experiments performed on A*β*(1-42) solutions devoid of seeds, showed that A*β* assembly into amiloyd fibrils was specifically forced in the presence of GM1-containing liposomes^16^,^17^. Moreover, it was found that A*β*-GM1 binding is accelerated in the presence of cholesterol, by the generation of GM1 clusters^18^,^19^, as they are in lipid rafts. Some suggestive morphology of A*β*-membrane interaction is shown by microscopy, while structural aspects substantially rely on simulation description^20^,^21^, determining the molecular sites involved in A*β*-GM1and A*β*-cholesterol interaction^22^,^23^. Also the notion that A*β* folding and aggregation route may be different whether free in a solvent or in the presence of a membrane is emerging^24^.

Recently, we have developped a model for lipid rafts suitable for structural investigation by neutron reflectometry^25^. We prepared and characterized lone macroscopic membranes (nanometer single bilayer thickness, centimeter lateral extension) with biosimilar composition in phospholipid, GM1 and cholesterol and, notably, with asymmetric distribution of components, a distinctive property of cell membrane rafts. The neutron reflectometry technique enables revealing the transverse structural details of a bilayer, discriminating regions at different depths within the membrane, avoiding radiation damage. Moreover, the contrast variation strategy^26^, exploiting the different cross-section of Hydrogen and Deuterium nuclei to a neutron beam, allows to enhance the visibility of an H-containing peptide in interaction with a fully-deuterated D-phospholipid membrane.

In the present study, we apply the neutron reflectometry technique to examine the interaction between a raft mime membrane and A*β*(1-42) oligomers. We focus on two conditions: 1) when the A*β*(1-42) reaches the raft-mime surface already in the membrane-active structured-oligomer state and 2) when early, unstructured A*β*(1-42) forms are administered and peptide oligomerization possibly takes place at the membrane. A good definition of the aggregation state of the peptide was allowed by the use of a non-aggregating precursor that can be activated to the native conformational state of A*β*(1-42), therefore avoiding the presence of preformed seeds and reducing structural polydispersity^27^,^28^,^29^.

We point out that thanks to the H-D neutron contrast enhancement, the use of fluorescent dyes at the N-terminus of the peptides, as commonly used in microscopy techniques^30^, can be avoided. The binding of fluorescent groups to this end of A*β* peptides is generally considered non-invasive^31^. Nonetheless, a modification of the specific properties of the N-terminus environment cannot be excluded. This portion has been recently proved to play a critical role in the A*β*(1-42) supramolecular assembly^27^. In fact, a point-mutation in this region (A2V), correlated to the early-onset AD only in homozygous carriers, was found to induce a prompter formation of structured oligomers and amyloid fibrils. Reversely, the co-incubation of the wild-type and the point-mutated A*β* gives rise to slowly-forming and labile aggregates^27^,^32^. A specific role, if ever, of the N-terminal portion in peptide-membrane interaction has not been defined. In the present study, we also tested the membrane-interaction ability of the N-terminal sequence of the A*β*(1-42) peptide, namely, the A*β*(1-6) fragment.

## Results and Discussion

The experiment was designed to study the existence, extent and structural details of the interaction between the membrane-active oligomeric species of A*β*(1-42) amyloid peptide and a raft-mimic membrane, containing ganglioside GM1 in the exposed leaflet.

To this scope, neutron reflectivity from a single macroscopic model membrane of customized composition and distribution of components is a powerful tool. In fact, it allows to describe the cross structure of the membrane, and how it is affected by the exposure to the peptide action, to the Ångstrom detail. In addition, the timescale is much longer than few-nanosecond, typical for molecular dynamics simulation, and the lateral extension (several cm^2^) allows for high statistical significance. Moreover, the use of fully deuterated phospholipids for the membrane matrix allows maximum visibility to incoming H-containing molecular species following exposure, when seen by neutron investigation. Any modification in the membrane structure involving H-recruitment or H-loss is immediately evident.

The raft-mime model membranes were built with biomimetic composition and, notably, with asymmetric cross distribution. They include ganglioside GM1, asymmetrically embedded only on one side of the membrane, and cholesterol that forms together with GM1, in typical mole fraction, a collective structural pair distinctive of rafts^25^. GM1, within rafts, is suggested to participate in A*β* interaction with the membrane and to act as a templating spot for A*β* aggregation^16^. Being the focus on the different peptide species, the long-chain DSPC was used as a matrix-phospholipid, as it forms very stable membranes, and the adhering-membrane configuration was chosen in order to ensure at best resistance to mechanical action during experimental manipulation.

### The target raft-mime membranes

**Figure 1:**
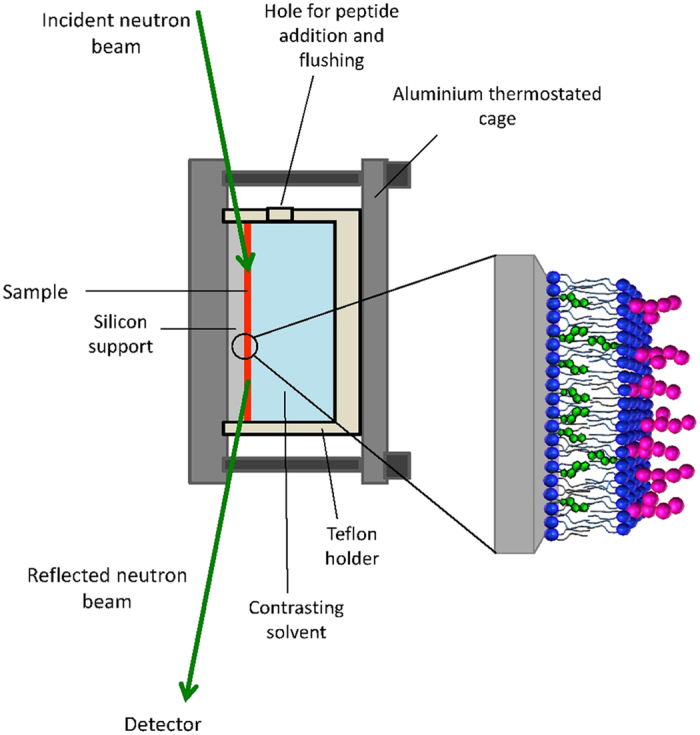
Scheme of the neutron reflectometry experimental set-up.

Three supported membranes (A, B and C) were independently prepared, each one with the raft-mime phospholipid:cholesterol:GM1 ganglioside composition (outer leaflet 10:0.74:1 – inner leaflet 10:1.76:0)^25^. Neutron reflectivity measurements, on each single hydrated membrane, were carried out at 22 °C in the experimental configuration sketched in Fig. 1.

Membranes were fully characterized in three contrast-solvents: H_2_O, D_2_O and a H_2_O/D_2_O mixture with scattering length density 4*10^−6^ Å^−2^ (4 MW), before being exposed to the A*β* peptides (See Materials and Methods section). This procedure, followed by the simultaneous fit of the three reflectivity curves, allows for the univocal identification of the physical parameters of the membranes. Unavoidably, each membrane is unique, nonetheless the three showed up to be nicely similar.

**Figure 2:**
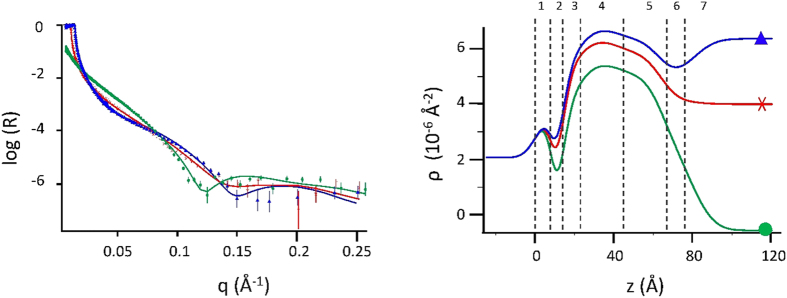
Multiple-contrast analysis of Membrane A at 22 °C. Spectra (left panel) and contrast profiles (right panel) in: H_2_O (green dots), D_2_O (blue triangles) and 4 MW (red crosses). *Left panel*: symbols mark the experimental spectra, lines the multi-contrast combined fit. R is the normalized reflected intensity. *Right panel*: over contrast profiles, vertical dashed lines are drawn to guide the eye to approximately identify 7 regions, referring to different portions of the reflecting system: the silicon oxide (*1*), a water layer (*2*), the inner hydrophilic layer (*3*), the inner hydrophobic layer (*4*), the outer hydrophobic layer (*5*), the outer hydrophilic layer (*6*) and the bulk solvent (*7*).

Figure 2 shows the experimental reflectivity spectra of membrane A in the three contrast-solvents, together with the corresponding multiple-contrast fit (left), and the obtained scattering length density profiles (right). Vertical dashed lines roughly identify the different regions of the supported membrane corresponding to the six layers of the fitting model (See Materials and Methods). The region marked *7* is that of the bulk solvent. The contrast profiles are different, as the scattering length densities of the three solvents are very different. This is particularly evident in regions 6 and 7, where the amount of solvent is significant, or substantial. Nonetheless, the physical parameters of the membrane are the same, as expected. From the contrast profiles in Fig. 2, it is also easily seen that highest membrane visibility is achieved in H_2_O. In the following, only the contrast profiles in H_2_O will be shown, for clarity, with the same nomenclature and numbering (*1–7*) as in Fig. 2.

**Figure 3:**
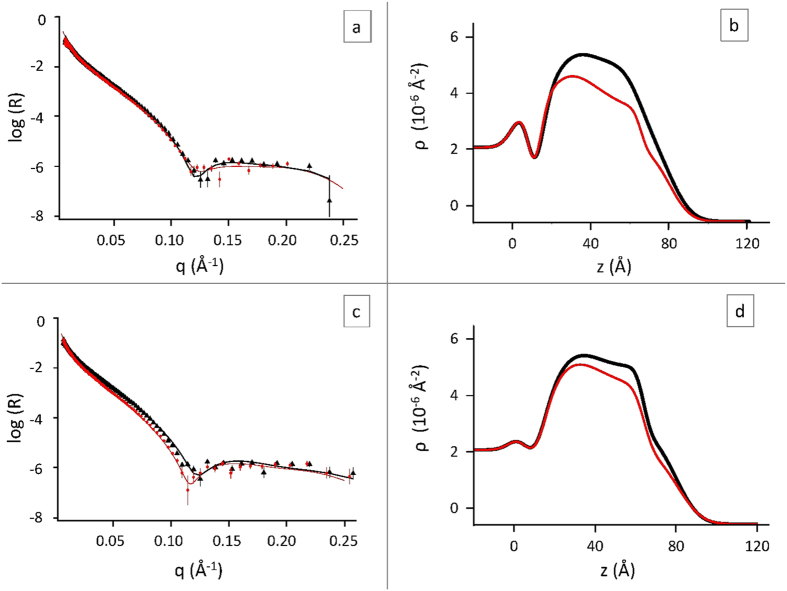
Neutron reflectivity spectra of membranes A and B before (black, triangles) and after (red, dots) the interaction with A*β*1-42 structured-oligomers (panel **a**) and early-oligomers (panel **c**) respectively, in H_2_O at 22 °C. Lines are the best fit to the experimental data. Panel (**b**) and panel (**d**) report the contrast profiles corresponding to spectra in panel (**a**) and panel (**c**), respectively (same color code).

**Figure 4:**
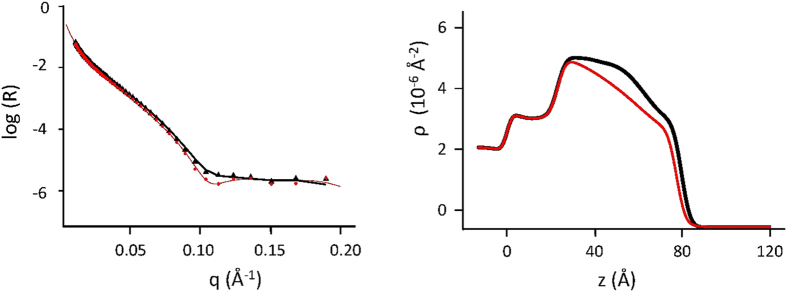
Neutron reflectivity spectra (left panel) and contrast profiles (right panel) of membrane C before (black, triangles) and after (red, dots) the interaction with the N-terminus A*β*1-6, in H_2_O at 22 °C.

The spectra relative to the three target membranes and their corresponding profiles are reported in Figs 3 and 4 (thin green lines, triangles), in comparison with their analogues after exposure to A*β* peptides. Their structural parameters are reported in Tables 1, 2 and 3 (left blocks).

### Target raft-mime membranes after the interaction with active A*β*(1-42) oligomers

Neutron Reflectivity was measured for both A and B membranes after exposure to the A*β*(1-42) peptide (See Materials and Methods section).

During injection into the sample holder, oligomers were dispersed into the solvent in contact with the exposed membrane. We have already shown^29^,^33^ that A*β*(1-42) structured-oligomers (prepared at 100 μM and incubated in phosphate buffer, 50 mM for 5 hours at 22 °C) are stable structures, resistant to dilution, both 1:10 and 1:100. Reversely, A*β*(1-42) early-oligomers are labile upon similar dilution, producing particles with hydrodynamic diameter much lower than 10 nm, easily monomers.

Figure 3 shows that interaction occurred between the membrane and A*β*(1-42), both in the form of structured-oligomers (top panels, membrane A) and of early-oligomers (bottom panels, membrane B). The reflectivity spectra (panels a and c) and the contrast profiles of the membrane (panels b and d) have been modified. The lowering in contrast shows that H-recruitment has occurred, originated from either A*β*(1-42) or water income. No similar effect was observed for the membrane left in contact with pure solvent, even upon extensive flushing, indicating that it occurs due to interaction with A*β*(1-42).

The scattering length density profiles of Fig. 3 reflect the distribution of admixed components within the membranes. Results are summarized in Tables 1 and 2, reporting the structural parameters of the target membranes A and B before (left block) and after exposure to A*β*(1-42), either structured-oligomers or early–oligomers, respectively. We first observe, from profiles and tables, that the overall thickness of both membranes is kept, indicating that, in both cases, interaction is not merely peripheral, i.e., surface adhesion or external carpeting. Intimate interaction has occurred. In both cases, two alternative extreme hypotheses have been considered. The first assumes that H-recruitment comes from A*β*(1-42) income (central block in Tables 1 and 2), the second that it comes entirely from water penetration (right block in Tables 1 and 2), involving membrane destabilization and loss of lipids. We recall that membrane destabilization, induced by the interaction of lipids with A*β* in its different aggregate forms, has been reported to occur and to be selective, bringing to the release of membrane lipids^34^. In the following we present and discuss separately the effect of A*β*(1-42) structured oligomers and early oligomers.

### Raft-mime membrane interaction with Aβ(1-42) structured oligomers. Membrane filling

We first address the most acknowledged active A*β* species, that is, the structured oligomers (See Materials and Methods section) From Fig. 3 and Table 1, we see that, after interaction with A*β*(1-42) structured oligomers, all partial membrane thicknesses are kept, but the membrane chemical composition is modified down to the midplane. Also the roughness at the midplane has increased, from 9 to 15 Å, showing that hydrophobic penetration is deep and effective, likely disturbing the disposition of cholesterol within the membrane. An effect of cholesterol on the midplane roughness has in fact been observed in similar model membranes^35^. On the other hand, we observe that the other interfacial roughnesses are not appreciably affected.

**Figure 5:**
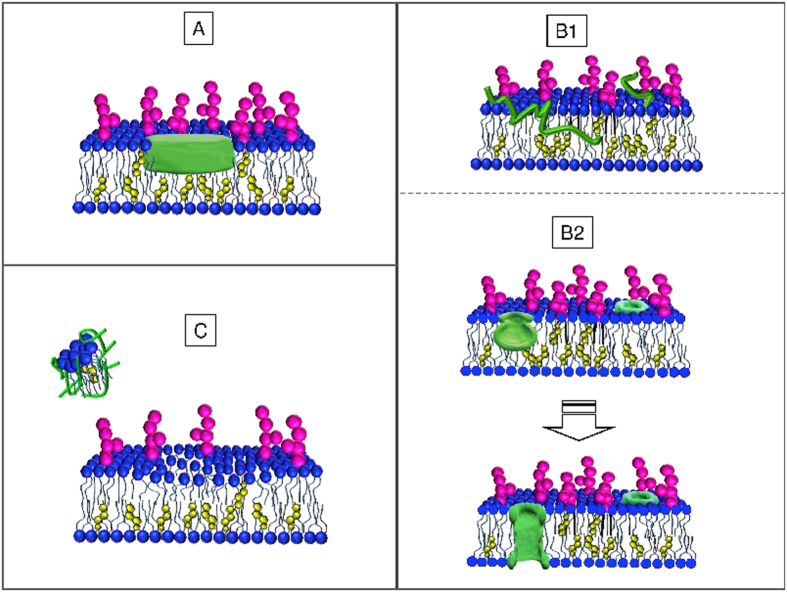
Pictorial sketch of a biomimetic membrane interacting with A*β* peptides: (**A**) A*β* peptides in the membrane-active structured-oligomer state (Membrane filling); (**B**) A*β* peptides in the monomeric state: (**B1**) monomers embroider the membrane and (**B2**) peptide oligomerization takes place next to membrane surface (Membrane digging); (**C**) membrane interacts with the N-terminal Aβ1-6 sequence (Membrane leakage).

Let’s focus on the central block of Table 1, relative to the hypothesis that A*β*(1-42) structured-oligomers recruitment has occurred. The relative reduction in contrast of the original hydrophobic and hydrophilic volumes of the outer membrane leaflet, following exposure, suggest that the structured-oligomers, sketched as discoidal particles inserting into the membrane, span the whole 17 Å thickness of the heads region and dive ∼12 Å in the tail region, covering an overall distance from the membrane surface of ∼29 Å, without appreciable protrusion. Besides assessing the occurrence and extent of interaction of the structured-oligomers with the membrane, this experiment allows to estimate the thickness of A*β*(1-42) structured oligomers, ∼30 Å. This value falls in the range of thickness for structured-oligomers proposed in the literature^36^. The membrane-filling model for the interaction of A*β*(1-42) structured-oligomers with the membrane, is sketched in Fig. 5A. The preservation of membrane thicknesses upon interaction and the agreement with literature data on oligomer size makes this model more likely than pointing at membrane destabilization and lipid loss (right block in Table 1).

The membrane-filling model, although showing some similarity to the carpeting effect proposed in the literature^37^, clearly indicates a non-peripheral interaction with the membrane components. Once in the membrane, structured-oligomers can constitute a seed for further adhesion of monomers and oligomers and elongation towards fibrillar structures.

### Membrane interaction with early oligomers. Membrane digging

In the following we address the A*β* early-oligomers. During injection into the measuring cell, early-oligomers were dispersed into the solvent in contact with the exposed membrane, undergoing a 40-fold dilution. Opposite to structured oligomers, A*β*(1-42) we have shown that early-oligomers^29^,^33^ (prepared at 100 μM and immediately diluted to 1 μM) promptly disaggregate upon dilution into particles with hydrodynamic diameter definitely much lower than 10 nm, likely mainly into monomers. So, in this part of the experiment, A*β*(1-42) reaches the membrane surface in the predominant form of monomers. We point out that, with this experimental procedure, we possibly mime the situation encountered by an individual A*β*(1-42) sequence freshly cut, by β- and γ-secretase enzymes, from its APP parent protein, while protruding from the surface of a membrane. The monomer, just freed from its APP chemical anchor, likely happens to be closer to the membrane than to other similar fragments, and, if not repelling, the membrane easily constitutes its first interaction site. Further A*β*(1-42) aggregation, if ever, occurs under the membrane constraints, presumably very different from those found in the bulk solution. In the literature, A*β* aggregation is claimed to be membrane-driven and, in particular, GM1, which is asymmetrically included in the outer leaflet of our cholesterol-containing raft-mime target membranes, is suggested to play a role as a template for its aggregation^38^,^39^.

From Table 2, we see that all partial membrane thicknesses are kept, but the membrane modification following interaction with A*β*(1-42) early-oligomers has been more extensive than for structured-oligomers. In fact, it involved the external lipid layer, down to the midplane of the membrane, and also below, in the inner hydrophobic region and disturbing the interface with the inner heads, increasing its roughness.

After interaction with A*β*(1-42) early-oligomers/monomers, the membrane composition showed a peculiar modification. A major H-recruitment occurred in the hydrophobic portion of the external leaflet, whereas the external hydrophilic region was less affected. A sole lipid loss, replaced by water, is unlikely, as it should induce roughly uniform changes in the same leaflet of the membrane. Cholesterol redistribution cannot correct this profile modification. We recall that the target membrane is very stiff, as it is made of DSPC, with (C18)-gel-chains, GM1, with ceramide-chains, and cholesterol. This composition, chosen both for stability and biosimilarity, is also likely to be very hard to penetrate. In fact, we see that in this case the affinity of A*β*(1-42) with the membrane core is high, and permeation is deep.

The hydrophobic : hydrophilic balance of A*β*(1-42) residues is 60 : 40, consistent with the fractional hydrophobic : hydrophilic substitution in the exposed membrane, (6 + 42) : 32 (see table 2). One could then sketch the membrane as embroidered by the A*β*(1-42) peptide, as in Fig. 5B1.

The peculiar H-recruitment profile, nonetheless, suggests another interesting as in Fig. 5B2. There, we sketch A*β*1-42 monomers undergoing aggregation within the templating structure of the biomimetic membrane, proceeding from its external side towards the opposite one. The jug-shaped profile draws the frontline, puncturing being not fully reached on the considered delays or, maybe, arrested by the presence of the solid silicon support. Some water dragging could also be involved. Membrane puncturing is claimed as one of the negative effects operated by A*β* oligomers^4^ sometimes directly observed by EM^5^. The picture sketched in Fig. 5B2 is also consistent with the AFM observation, once the peculiarities of the experimental methods are properly accounted for. This membrane-digging model is highly appealing, as it stems for a true structural interference between the raft-mime membrane and the organizing peptide. The influence of a hosting lipid aggregate on the A*β* folding has been addressed in the literature, GM1 ganglioside playing a prominent role^40^.

We observe that the present results seem to be partially contrasting with those obtained by single-molecule imaging techniques^30^, exploring the initial interactions between A*β* monomers and oligomers and the membranes of living cells. In fact, while concluding that the oligomers become immobilized on the cell surface, they find that oligomers preferentially interact with cell membranes, relative to monomers. Nonetheless, we also underline that the single-molecule imaging experimental technique, although very sensitive, requires peptide labeling that was operated in the N-terminal region of A*β*. As we discuss in the following, and differently from what generally assumed, also this region could be involved in the peptide-membrane interaction, thus suffering from label-group bias.

### Membrane interaction with the N-terminal Aβ(1-6) sequence. Membrane leakage

Neutron Reflectivity was measured for membrane C after exposure to the A*β*(1-6) terminal sequence of A*β* (See Materials and Methods section). This portion of A*β* is not commonly assumed to participate in the interaction with membranes. Its small size coupled with its aminoacidic composition (with two acid, two basic, two hydrophobic residues) does not claim for a clear propensity to enter the membrane, at first sight, and the N-terminus sites involved in GM1 interaction reside in a successive portion of A*β*^22^. The peptide A*β*(1-6) itself, differently from the highly amyloidogenic A*β*(1-42), preserves its monomeric form and does not display any tendency to aggregate. Moreover, as indicated by CD analysis (See Supporting Information), the short peptide Aβ(1-6) presented an unordered secondary structure that did not change over time. In fact, the spectra at 72-hours incubation at 37 °C showed the same signal recorded at zero time (Fig. S1). This indicates no propensity to aggregate and high stability in solution. Nonetheless, some involvement in A*β* evolution has been recently identified. It has been shown that the first amino acid residues at the N-terminal of A*β* play an important role in the formation of stable β-sheets in the secondary structure^41^. Besides, it has been shown that a point modification at position 2, in this region, affects the kinetics, extent and stability of aggregates, starting from the early stages of oligomerization, indicating that the N-terminus of A*β* is involved in the aggregation process^27^.

Aim of this part of the experiment was to test if any propensity is shown by the A*β*(1-6) N-terminus to an interaction whatever with the membrane. Figure 4 clearly shows that interaction between A*β*(1-6) and the membrane has occurred. Peripheric adhesion of the peptide to the membrane, connected to electrostatic interaction that could have resisted to water washing, was excluded, as superimposable results were obtained after rinsing again with a 156 mM NaCl solution. The lowering in contrast, as in Fig. 4, shows that H-recruitment has occurred, involving the external lipid layer down to the midplane of the membrane, clearly increasing its roughness. Table 3 reports the structural parameters of membrane C before (left block) and after exposure to the A*β*(1-6), in the same two alternative extreme hypotheses, as before.

In this case, the water-penetration hypothesis, right block of Table 3, results in the same fractional volume being replaced in the hydrophilic and hydrophobic moieties of the external membrane layer (13%). This result strongly stems for whole-lipid extraction operated by A*β*(1-6). Interstingly, an overall 3/40 (7.5%) thickness reduction of the outer layer is also observed, as for a self-healing action following lipid loss (∼20%). Considerable lipid loss and membrane remodeling is also reflected into the evident roughness increase.

We can then hypothesize a specific role for the N-terminus of A*β* in membrane leakage, promoting A*β* peptide entry. In fact, a mechanism of lipid removal, maybe lipid-selective, has been proposed as partly responsible in the overall toxic action of A*β* oligomers^34^, in line with the present results.

## Conclusion

The concept that A*β* oligomeric species play a fundamental role in the development of Alzheimer disease is widely accepted, attributed to their membrane-active features. Nonetheless, several processes are hypothesized, connected to different suggestions coming from biochemistry, microscopy and simulation experiments. Population unbalance, favouring either labile or structured oligomers, is a fascinating route, yet the designation of the more effective species in promoting the disease, if ever, is not clear. This is a topic of importance when, besides understanding the basic phenomena, a therapeutic strategy is pursued, to prevent progression or promote regression of the disease.

In this neutron reflectivity study, we investigated the interaction of A*β* with a single asymmetric complex membrane, containing cholesterol and monosialoganglioside GM1, a good experimental model for lipid rafts, putative sites for A*β* settling and seeding.

We conclude that, both claimed membrane-active species of A*β*, namely early-labile and structured oligomers, interact with the membrane, their association being not peripheral nor purely electrostatic. Nonetheless, differences exist in the extent and depth of interaction, interestingly pointing at unexpected relative impacts on the membrane We observed that structured oligomers are embedded as such in the outer leaflet of the membrane. There, they can constitute a seed for further A*β* addition and elongation. Reversely, we found that early labile oligomers, easily dissolving to monomers, are captured by the membrane and deeply dig it towards the opposite side. An eventual deeper impact of monomers as compared to oligomers is surprising, based on current concepts. A peculiar profile suggests that A*β* organization, starting from enclosed monomers, is templated by the membrane into a forming pore. Furthermore, we hypothesize a role for the 1-6 N-terminal sequence of A*β*, namely in membrane destabilization, then facilitating A*β* recruitment. Mutations in the N-terminal sequence could then be more effective than anticipated in modulating both amyloid aggregation promptness and stability and amyloid interaction with cell membranes.

## Materials and Methods

Deuterated 1,2-distearoyl-sn-glycero-3-phosphatidylcholine (d_83_-DSPC) was purchased from Avanti Polar Lipids. Cholesterol was purchased from Sigma-Aldrich Co. and GM1 (Neu5Acα2-3(Galβ1-3GalNAcβ1-4)Galβ1-4Glcβ1Cer) was extracted and purified as described in Ref. 42 and obtained as sodium salt powder. Synthetic A*β*1-42 (DAEFRHDSGYEVHHQKLVFFAEDVGSNKGAIIGLMVGGVVIA) and A*β*1-6 (DAEFRH) peptides were prepared on a 433 A synthesizer (Applied Biosystems, Foster City, CA) using a solid-phase peptide synthesis (SPPS) with Fmoc chemistry^43^. A*β* was synthesized using depsipeptide method^44^. Peptides were cleaved from NOVASYN-TGA resin^45^ and purified by reverse phase HPLC on a semi-preparative jupiter C4 column (300 Å, 10μm, 250 × 21.2 mm, Phenomenex) using water : acetonitrile gradient elution. Peptides identity was confirmed by MALDI-TOF analysis (Reflex III, Bruker) and their purity was above 90–95%^43^. The depsipeptide technique is specific for so called difficult peptides, such as *β*-amyloid, because it allows to obtain a batch with a low degree of aggregation, free of either highly folded structures or fibrils and aggregates, as much as possible near to monomer condition.

Aliquots from the same batch of depsi-A*β*(1-42) were stored in acidic solution (TFA 0.02%) at not less than 200 μM concentration and the native sequence was obtained following the switching procedure in basic condition^27^. The peptide solution was then diluted to 40 μM in 50 mM phosphate buffer, 150 mM NaCl, pH 7.4, and used after five minutes (A*β*(1-42) early-oligomers) or incubated for 4 hours at 22 °C (A*β*(1-42) structured-oligomers). A*β*(1-6) was dissolved in water to a 200 μM concentration, then diluted to 100 μM in phosphate buffer, pH 7.4, 150 mM NaCl (PBS). This procedure allows to reduce structural polydispersity and avoid the presence of seeds with different structural features and compactness^33^.

### Raft-mime bilayer build-up

Samples were deposited via Langmuir films of desired composition, by the Langmuir-Blodgett and Langmuir-Schaefer techniques^46^,^47^. Cholesterol, d_83_-DSPC and GM1 ganglioside were individually dissolved in the appropriate organic solvent (chloroform or chloroform : methanol = 2 : 1) to a final concentration of 1 mg/ml. Mixed lipid systems were obtained by mixing appropriate amounts of single-lipid solutions. Substrates were single crystals of silicon (5 × 5 × 1.5 cm^3^) polished on one large face (111), cleaned before use in appropriate organic solvents and treated with UV-Ozone for 30 min^48^. Langmuir depositions were carried out on a Langmuir trough (NIMA, UK), filled with pure water and kept at T = 15 °C. Before deposition, each monolayer was compressed to a surface pressure of 40 mN/m, and then the protocol described in Ref. 25 was followed. d_83_-DSPC, was chosen for the bilayer matrix for its stability and compactness^49^. Measurements were carried out at 22 °C, where d_83_-DSPC lipid chains are in the gel phase. The choice of a fully deuterated phospholipid matrix, allowed enhancing the visibility of the peptides, containing hydrogen atoms, in interaction with different regions of the floating membrane.

Three raft-membranes were independently prepared, with the same composition. Their overall composition is d_83_-DSPC : cholesterol : GM1 = 10 : 1.25 : 0.5, a mole ratio similar to that of rafts. Also the asymmetric disposition of components mimics the one naturally occurring in rafts, being the composition of the inner leaflet d_83_-DSPC : cholesterol : GM1 = 10 : 2 : 0 and that of the outer leaflet d_83_-DSPC : cholesterol : GM1 = 10 : 0.5 : 1, according with the components distribution found in Ref. 25. Unavoidably, each membrane is unique, nonetheless, as shown in the Figures and Tables, the three showed up to be nicely similar. Membrane C was slightly different from membranes A and B, which were actually prepared during a different experiment. Lipid adhesion during preparation could in fact be affected by, for example, the silicon oxide layer on top of the silicon block, or random environmental factors.

### Membrane exposure to A*β* peptides

The three target raft-membranes were characterized by NR and then exposed to A*β* peptides. Membranes A and B were exposed to A*β*(1-42) structured-oligomers or early-oligomers, respectively. 50 μl of the two peptide preparations at 40 μM were injected directly into the measuring cell, in the bulk water, to a final concentration of 1 μM Aβ peptide and lipid:peptide = 3:1 molar ratio. During injection into the sample holder, oligomers were dispersed into the solvent in contact with the exposed membrane. We have already shown^29^,^33^ that A*β*(1-42) structured-oligomers (prepared at 100 μM and incubated in phosphate buffer, 50 mM for 5 hours at 22 °C) are stable structures, resistant to dilution, both 1:10 and 1:100. Reversely, A*β*(1-42) early-oligomers are labile upon similar dilution, producing particles with hydrodynamic diameter much lower than 10 nm, easily monomers. In both cases, the peptides were allowed to interact with the membranes for 30 minutes, followed by a first reflectivity measurement, then rinsed by slowly flushing H_2_O, and reflectivity was measured again. No detectable difference was observed between the reflectivity spectra collected before and after water flushing.

Membrane C was exposed to the peptide A*β*(1-6). 50 μl of the A*β*(1-6) solution prepared at 100 μM were added in the bulk water to a final concentration of 2.5 μM and a lipid : peptide = 1.2 : 1 mole ratio. A higher amount of A*β*(1-6) was injected, as compared to A*β*(1-42), as this N-terminus portion, not belonging to the hydrophobic domain of A*β*1-42, is normally expected to show a scarce or null propensity to interact with the membrane. A*β*(1-6) was allowed to interact with membrane C for 30 minutes, followed by a first reflectivity measurement, then rinsed by slowly flushing H_2_O, and reflectivity was measured again. Finally, the sample was rinsed with NaCl 156 mM solution, to screen electrostatic interactions, and reflectivity was measured again. No detectable difference exists in the reflectivity profiles obtained before and after water and buffer flushing.

### Neutron Reflectivity Measurements and Data Treatment

In a neutron reflectivity experiment, the intensity of a neutron beam reflected by a surface is measured as a function of the momentum transfer perpendicular to the surface itself^50^. For stratified samples, like membranes, information about their internal structure can be obtained. The sample is seen as a series of parallel thick interfaces, each one reflecting, and transmitting, the incident beam according to its “optical contrast”, depending on its chemical composition. Namely, the scattering length density ρ of a composite material with *n*_*j*_number of nuclei per unit volume is given by *ρ *=* Σ*_*j*_ *b*_*j*_*n*_*j*_ , where *b*_*j*_ is the scattering length of nucleus *j*. For stratified samples, the interference of the beams reflected by each internal interface is recorded. A detailed description of the cross structure of a membrane can be obtained, (both for supported^51^,^52^,^53^ and floating^25^,^35^,^54^,^55^ membrane systems) as well as its structural modification following its exposure to varied conditions or external agents. This potentiality is becoming exploited and pioneering work is being done addressing different topics, like the membrane response to an external electric field^56^ or to exposure to cationic vesicles^57^, enzymes^58^ or peptides^59^.

Reflectivity measurements were performed on the vertical-sample reflectometer D17 (TOF mode, 2 Å ≤ λ ≤ 20 Å, incident angles θ_1_ = 0.7° and θ_2_ = 4°) at ILL (Grenoble, F) at the silicon-water interface, the beam coming from the silicon block side. Measurements on a bare silicon substrate were performed to characterize the silicon oxide layer at the silicon surface.

For some of the reflectivity measurements, and when useful for data analysis, membranes were observed in the presence of three contrasting solvents, namely, H_2_O (Milli-Q) with a scattering length density of −0.56*10^−6^ Å^−2^, D_2_O (99% pure, ILL) with a scattering length density of 6.35*10^−6^ Å^−2^ and 4-Match Water (4 MW), that is, a mixture of H_2_O and D_2_O with a scattering length density of 4*10^−6^ Å^−2^. The multiple-contrast procedure allows for characterizing the deposited membrane with a high degree of confidence. Nonetheless, we underline that the use of this technique with mixed systems has to be carefully evaluated, because it requires subsequent washing with fresh solvent, a procedure that could preferentially and progressively remove species of higher solubility.

Data were analyzed using the software Motofit^60^ allowing to draw the cross profile of the sample membrane, that is, the contrast ρ(z), thickness and roughness of each of the internal layers. A 6-layer model was used: the silicon oxide (*1*), a water layer (*2*), the inner hydrophilic layer (*3*), the inner hydrophobic layer (*4*), the outer hydrophobic layer (*5*) and the outer hydrophilic layer (*6*). The chemical composition of each layer was then assessed by using, for its individual components, the appropriate scattering length density (SLD) values (see Supporting Information).

The best fits have been evaluated as the ones giving the minimum χ^2^ among all the good quality fits, which have been used to evaluate the errors on parameters values, reported on the footnotes of tables. As an example see Fig. S1 in the Supporting Information. In Tables 1, 2 and 3 the parameters coming from the best fits of the experimental data, represented in Figs 3 and 4, have been reported.

### Circular Dichroism

Secondary structure and stability of A*β*(1-6) peptide were determined using circular dichroism (CD) technique. A*β*(1-6) solutions were analyzed at zero time and after 72 hours of incubation at 37 °C. CD-spectra were recorded on a Jasco J-815 spectropolarimeter (Jasco, Easton, USA) in the range 260–190 nm using a 1 mm quartz cell. All spectra were performed with a band width 1.0 nm (0.1 nm resolution). All spectra come from an average of five scans with a sensitivity of 100 mdeg, a response of 4 sec and a scan speed of 50 nm/min. CD spectra,after buffer spectra subtraction. were expressed as mean molar ellipticity (Φ).

## Additional Information

**How to cite this article**: Rondelli, V. *et al.* Amyloid-β Peptides in interaction with raft-mime model membranes: a neutron reflectivity insight. *Sci. Rep.* **6**, 20997; doi: 10.1038/srep20997 (2016).

## Supplementary Material

Supplementary Information

## Figures and Tables

**Table 1:**
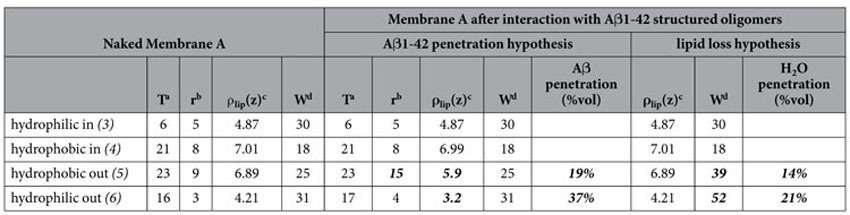
Physical parameters of Membrane A, before and after exposure to A*β*(1-42) structured-oligomers.

Physical parameters of Membrane A, before (left) and after exposure to Aβ1-42 structured-oligomers: the central block follows the assumption that membrane modification comes from Aβ recruitment; the right block follows the extreme assumption of massive water penetration, with loss of lipids.

Numbering of membrane regions refers to that of Fig. 2. Significative changes in structural parameters are marked in bold.

^a^T: layer thickness (±1 Å).

^b^r: roughness between one layer and the adjacent previous one (±2 Å).

^c^ρ_lip_(z): average scattering length density of the lipid (non-water, non-peptide) components of the layer (±0.05*10^−6^ Å^−2^).

^d^W: percent water content of the layer (±5% in volume).

**Table 2:**
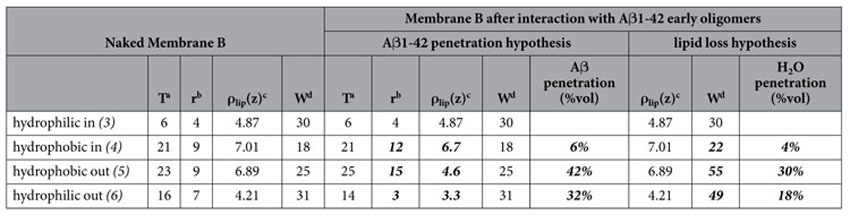
Physical parameters of Membrane B, before and after exposure to A*β*1-42 early-oligomers.

Physical parameters of Membrane B, before (left) and after exposure to A*β*1-42 early-oligomers: the central block follows the assumption that membrane modification comes from A*β* recruitment; the right block follows the extreme assumption of massive water penetration, with loss of lipids.

Numbering of membrane regions refers to that of Fig. 2. Significative changes in structural parameters are marked in bold.

^a^T: layer thickness (±1 Å).

^b^r: roughness between one layer and the adjacent previous one (±2 Å).

^c^ρ_lip_(z): average scattering length density of the lipid (non-water, non-peptide) components of the layer (±0.05*10^−6^ Å^−2^).

^d^W: percent water content of the layer (±5% in volume).

**Table 3:**
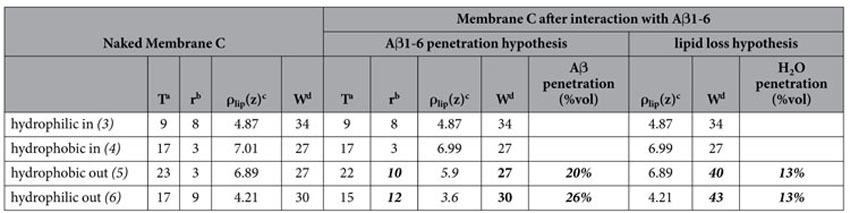
Physical parameters of Membrane C, before and after exposure to A*β*(1-6).

Physical parameters of Membrane C, before (left) and after exposure to A*β*1-6: the central block follows the assumption that membrane modification comes from A*β* recruitment; the right block follows the extreme assumption of massive water penetration, with loss of lipids.

Numbering of membrane regions refers to that of Fig. 2. Significative changes in structural parameters are marked in bold.

^a^T: layer thickness (±1 Å).

^b^r: roughness between one layer and the adjacent previous one (±2 Å).

^c^ρ_lip_(z): average scattering length density of the lipid (non-water, non-peptide) components of the layer (±0.05*10^−6^ Å^−2^).

^d^W: percent water content of the layer (±5% in volume).
