# Supplementary material for: Amyloidβ Peptides in interaction with raft-mime model membranes: a neutron reflectivity insight
Source: Sci Rep. 2016 Feb 16;6:20997. doi: 10.1038/srep20997 (PMC4754687; doi:10.1038/srep20997)
Supplement: Supplementary Information [file srep20997-s1.pdf]

Supplementary Information

Amyloid‑β Peptides in interaction with raft-mime model membranes: a neutron reflectivity insight.

Valeria Rondelli, Paola Brocca, Simona Motta, Massimo Messa†, Laura Colombo†, Mario Salmona†, Giovanna Fragneto‡, Laura Cantu'*, Elena Del Favero

Dept. of Medical Biotechnologies and Traslational Medicine, University of Milano, LITA, Via F.lli Cervi, 93. 20090 Segrate (Milano), Italy.

†Dept. of Molecular Biochemistry and Pharmacology, IRCCS Istituto di Ricerche Farmacologiche "Mario Negri", Via La Masa 19, 20156 Milan, Italy.

‡InstitutLaue-Langevin, 71 avenue des Martyrs, BP 156, 38000 Grenoble Cedex, France

Corresponding author:

Laura Cantu'

laura.cantu@unimi.it

Table S1: Theoretical SLD values of the individual chemical species

Compound	SLD [a]	Compound	SLD [a]	Compound	SLD [a]
H_2O	-0.56	DSPC d-chains	7.91	Cholesterol	0.22
D_2O	6.35	DSPC d-heads	4.87	Aβ1-42	1.50
Si	2.07	GM1 chains	-0.41	Aβ1-6	1.75
SiO_2	3.41	GM1 heads	2.23	4-Match Water	4

[a] SLD values (10^{-6}Å^{-2}) for lipids have been calculated from Refs s1, s2 and s3, and for Aβ peptides from Ref. s4 and assuming a density of 1.5 g/cm^3

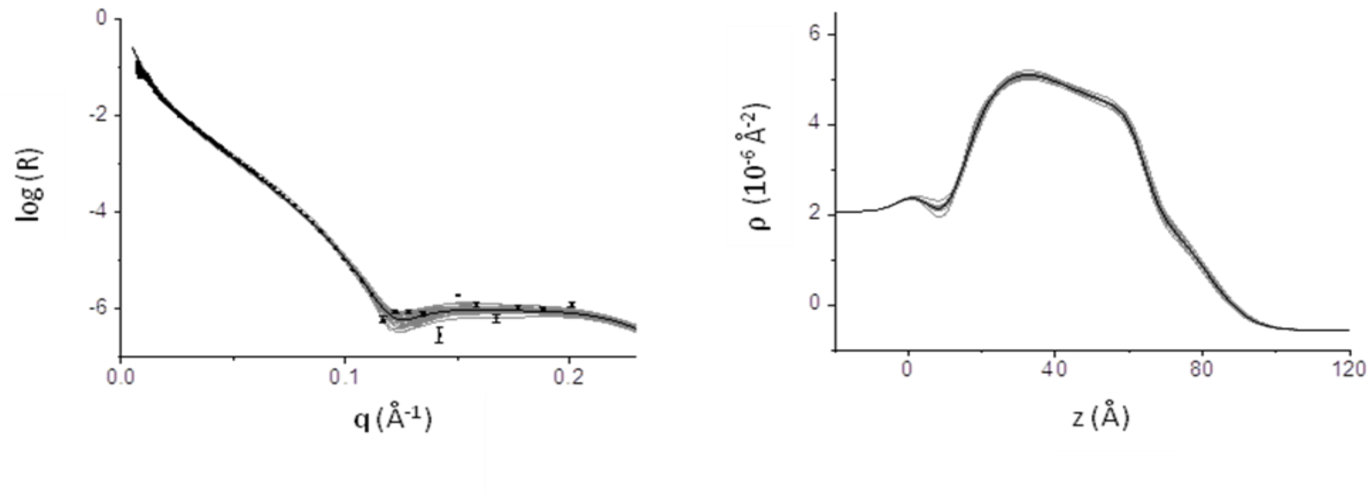

Figure S1. Neutron reflectivity spectrum (left panel) and contrast profile (right panel) of membrane A after the interaction with Aβ1-42 structured-oligomers in H_2O at T = 22°C. In the ***left panel*** dots are the experimental points. In black the best fit, giving the minimum χ^2, is reported, together with the curves, in grey, obtained by varying the fitting parameters within the errors. Errors on the fitting parameters, reported in the footnotes of Tables 1, 2 and 3 in the manuscript, have been estimated by varying each parameter until the quality of the fit was no more acceptable. On the ***right panel*** the contrast profile coming by the best fit is reported in black, together with the contrast profiles corresponding to the grey curves of left panel, thus representing a sort of error band to the best contrast profile.

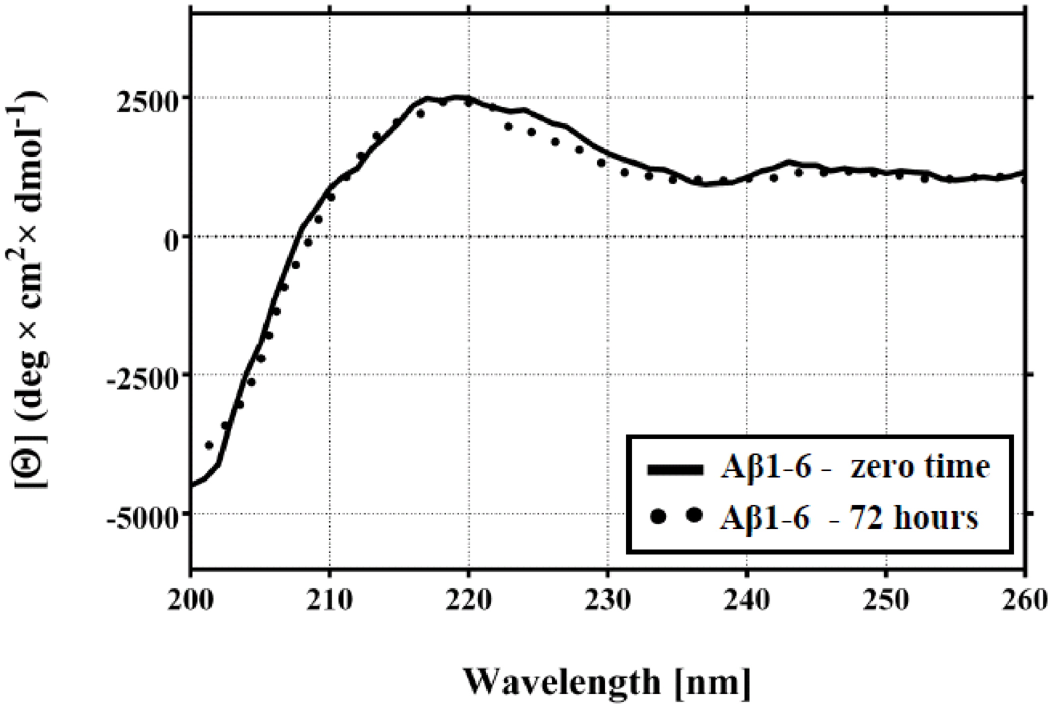

Figure S2. Secondary structure analysis of Aβ1-6. 100 μM Aβ1-6 peptide in 50 mM phosphate buffer containing 150 mM NaCl, pH 7.4, were analyzed at zero time and after 72-hours incubation. CD analysis showed that Aβ1-6 did not form β-sheet secondary structure, suggesting a no aggregation propensity and a high stability in solution

References

(s1) Nagle, J. F.; Wiener, M. C. Relations for Lipid Bilayers. *Biophysical Journal* **1989**, *55*, 309–313.

(s2) Greenwood, A. I.; Tristram-Nagle, S.; Nagle, J. F. Partial Molecular Volumes of Lipids and Cholesterol. *Chemistry and physics of lipids* **2006**, *143*, 1–10.

(s3) M. Boretta, L. Cantu, M. Corti, E. D. F. Cubic Phases of Gangliosides in Water: Possible Role of the Conformational Bistability of the Headgroup. *Physica* **1997**, *236*, 162–176.

(s4) http://it.sw3c.com/chemical/properties/cas-107761-42-2.html
